# DIMENSIONALITY, PROXIES, AND PREDICTORS OF COGNITIVE RESERVE IN THE MANCHESTER LONGITUDINAL STUDY

**DOI:** 10.1093/geroni/igae098.3604

**Published:** 2024-12-31

**Authors:** Stephen Aichele

**Affiliations:** Colorado State University, Fort Collins, Colorado, United States

## Abstract

In a sample of N=113 older adults (ages 62-86 years at MRI scan; 58.4% women) from the United Kingdom, we compared the dimensionality of general cognition before (COG) vs. after (COGr; cognitive reserve) residualization on global brain status (as indicated by brain volume loss, cerebral blood flow, and white matter lesion prevalence). Cognitive performance was assessed by 15 tasks spanning five domains: processing speed, fluid intelligence (e.g., spatial reasoning), crystallized intelligence (vocabulary), visuospatial memory, and verbal memory. Statistical tests were conducted in a series of structural factor analyses with progressive constraints on measurement invariance. Results showed that although COG and COGr were highly correlated (r =.84), item-factor loadings differed such that COGr more strongly emphasized fluid intelligence. We further examined differential associations of COG and COGr with 23 socio-demographic, health, and lifestyle variables in a series of regression models. Results from these latter tests showed consistent associations of education level, occupational class, and hobbies/interests as proxies both for COG and COGr. Difficulty with personal mobility was a salient risk factor for both COG and COGr, but only in models unadjusted for chronological age. In sum, results supported the role of intelligence as a potential compensatory factor for brain-related reductions in processing speed and memory. Efforts to preserve personal mobility and a diversity of hobbies and interests may serve as protective factors against brain-related reductions in cognitive performance.

